# Chronic physical illness in early life and risk of chronic widespread and regional pain at age 68: evidence from the 1946 British birth cohort

**DOI:** 10.1097/j.pain.0000000000000663

**Published:** 2016-07-14

**Authors:** Stella G. Muthuri, Diana Kuh, Rebecca Bendayan, Gary J. Macfarlane, Rachel Cooper

**Affiliations:** a MRC Unit for Lifelong Health and Ageing, UCL, London, United Kingdom; b Epidemiology Group, Institute of Applied Health Sciences, School of Medicine, Medical Sciences and Nutrition, University of Aberdeen, Aberdeen, United Kingdom; c Aberdeen Centre for Arthritis and Musculoskeletal Health, University of Aberdeen, Aberdeen, United Kingdom

**Keywords:** Chronic pain, Ageing, Childhood illness, Birth cohort, Life course

## Abstract

This study aimed to examine the associations between serious illness in earlier life and risk of pain in old age using data from a large nationally representative British birth cohort, the Medical Research Council (MRC) National Survey of Health and Development (NSHD). Serious illness was defined as any experience of illness before age 25 requiring hospital admission of ≥28 days. Pain was self-reported at age 68, with chronic widespread pain (CWP) defined according to American College of Rheumatology criteria. Multinomial logistic regression was used to test associations of serious illness in early life with CWP, chronic regional pain (CRP), and other pain, with no pain as the referent category. Adjustment was made for sex, socioeconomic position, adult health status, health behaviours, and psychosocial factors. Of 2401 NSHD participants with complete data, 10.5% reported CWP (13.2% of women and 7.7% of men), 30.2% reported CRP, and 14.8% other pain. Compared with those with no history of serious illness, those who experienced serious illness in early life had a higher likelihood of CWP (relative risk ratio [RRR] = 1.62 [95% CI: 1.21-2.17]) and of CRP (RRR = 1.25 [95% CI: 1.01-1.54]) after adjusting for sex. In fully adjusted models, serious illness in early life remained associated with CWP (RRR = 1.43 [95% CI: 1.05-1.95]), but associations with CRP were attenuated (RRR = 1.19 [95% CI: 0.96-1.48]). There were no associations with other pain. These findings suggest that those who have experienced serious illness in earlier life may require more support than others to minimise their risk of CWP in later life.

## Abstract 2

Supplemental Digital Content is Available in the Text.

In a British birth cohort study, experience of serious illness in earlier life is associated with increased risk of chronic widespread pain at age 68.

## 1. Introduction

With continued improvements in biomedical therapies and public heath, a growing number of people who have experienced chronic illnesses in early life are now surviving into midlife and beyond.^^31,38^^ These people are vulnerable to a disproportionate burden of morbidity and disability during adulthood.^^4,20,21,35^^ With the increases in life expectancy of these and other higher risk groups, the characteristics of the adult population 65 years and older are becoming increasingly heterogeneous. Understanding the long-term impact of chronic childhood illnesses is necessary for planning effective health and social care for older people. It may also identify appropriate interventions that could prevent or alleviate secondary burdens arising from these conditions, and identify those who require more support than others to maintain their quality of life in old age.

Population-based longitudinal studies of considerable size that have followed adult survivors of chronic childhood illness have been used to examine the impact of specific conditions, eg, asthma,^^36^^ childhood cancer,^^29^^ and congenital heart disease^^16^^ in young to middle adulthood, but few have examined a wide range of early chronic illnesses. These include the 2 oldest British birth cohorts which linked childhood illness to poorer physical health^^21,35^^ and physical disability in early adult life^^20^^; whether these associations extend into old age has not yet been established.

Pathways that link chronic physical illness in earlier life and health in later adult life are likely to be varied and cumulative. Chronic illness (eg, pulmonary tuberculosis, congenital heart disease, polio, cerebral palsy etc.) may cause functional disturbances or permanent biological changes that directly increase susceptibility to chronic conditions, functional limitations, and disability throughout life. Serious illness during early life may also indirectly influence adult health by affecting educational attainment, economic achievement, and social circumstances, and consequently influence health-related behaviour over the life course.^^32^^ Alternatively, it may increase the likelihood of psychosocial and psychiatric disorders, that can exacerbate chronic physical illness, which in turn may set up vicious cycles of worsening morbidity over the life course.^^34^^

Chronic pain is the most common morbidity in old age, and it seriously impacts on activities of daily living and quality of life of older adults.^^8,24,25,30^^ There have been few prospective investigations of the relationship between serious physical illness in earlier life and pain in adulthood, and results were inconsistent^^10,12^^; furthermore, none extend into old age.

**Figure 1.**
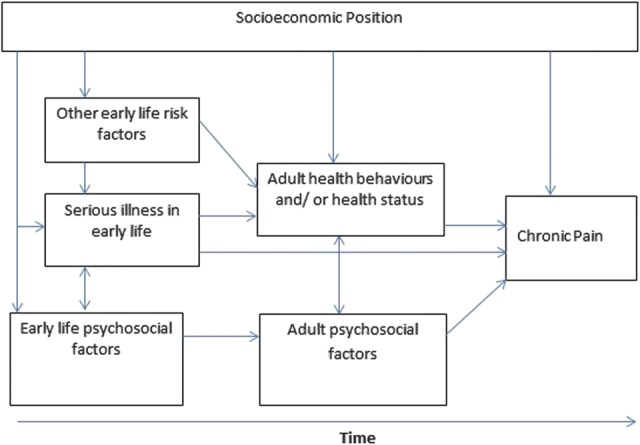
Potential pathways linking serious illness in early life and chronic pain at age 68.

Using data from the oldest British birth cohort, the Medical Research Council (MRC) National Survey of Health and Development (NSHD), we hypothesised that those who experienced serious physical illness in early life would be at increased risk of chronic widespread and regional pain in later life. We tested whether any associations observed were as follows: (1) independent of potential confounders, including sex, adult health status, and health behaviours, (2) indirectly associated with socioeconomic position through cumulative effects of low educational attainment and reduced employment opportunities over the life course, or (3) confounded by psychosocial factors (Fig. 1).

## 2. Methods

### 2.1. Study population

The NSHD is a population sample of 5362 births in England, Wales, and Scotland in 1 week of March 1946 that has been followed up 25 times.^^19,45^^ At age 68, 2943 eligible study members who were known to be alive and whose current contact information was known were sent a postal questionnaire. Of these, 2453 (84%) completed and 29 (1%) returned incomplete questionnaires. Of the remaining sample, 11 (0.4%) had died, 424 (14%) did not return the questionnaire, and 26 (0.9%) questionnaires were returned undelivered.

Ethical approval for the data collection at age 68 was obtained from the Queen Square Research Ethics Committee (14/LO/1073) and the Scotland A Research Ethics Committee, and written informed consent was obtained from study members.

### 2.2. Experience of serious illness in early life

Information on illnesses and injuries during early life, including hospital admission, age at admission, diagnosis, name of hospital, and length of hospital stay was obtained from mothers' reports across childhood and study participants' reports from age 18 up to 25 years. Any reported admissions were checked with the hospitals concerned and coded according to the International Classification of Diseases, Revision 8 (1965). We defined serious illness as any experience of physical illness before the age of 25 years that required hospital admission of at least 28 days (yes/no).^^20,21^^ Additional variables identified are included in the following: (1) the total number of periods during early life (ie, infancy [0-4 years], childhood [5-14 years], adolescence [15-19 years], or early adulthood [20-24 years]) that serious illness was experienced (range: 0 [no serious illness reported] to 4 [serious illness reported in all 4 periods of early life]); and (2) distinguishing potentially disabling illnesses (ie, polio [ICD8 codes: 040-046], diseases of central nervous system [320-349], mental retardation [310-315], circulatory diseases [400-458], musculoskeletal [710-738], congenital anomalies [740-759], and accidents [800, 810, and 820-860]) from other illnesses.^^20^^

### 2.3. Assessment of pain

In the postal questionnaire administered at age 68, participants were asked “*In the last month, have you had any ache or pain which has lasted for one day or longer*, *not including pain occurring during the course of a feverish illness such as flu?*” Those who reported pain were asked to: (1) indicate whether they had experienced this pain for at least 3 months and (2) shade the location of their pain using a 4-view body manikin. We then defined chronic widespread pain (CWP) according to American College of Rheumatology criteria for fibromyalgia^^46^^ as pain present for 3 months or longer, both above and below the waist; on both the left and right side of the body; and in the axial skeleton. Participants who reported chronic pain as present but did not meet the CWP definition were classified as having chronic regional pain (CRP), whereas those who reported acute (ie, pain in the last month that had lasted for less than 3 months) widespread or regional pain, or reported regional pain but chronicity was unknown (n = 30) were regarded as having “other” pain. Those who reported widespread pain but chronicity was unknown (n = 3) and those who did not indicate the site of pain (n = 8) were excluded from analyses.

### 2.4. Covariates

Potential confounders and mediators were identified a priori based on the existing literature and the framework outlined in Figure 1. These include specific behavioural risk factors (ie, higher body mass index (BMI),^^2,26,42^^ smoking,^^37,42^^ and physical inactivity^^5,27^^) and psychosocial factors (ie, adverse childhood events and experiences and current psychological distress)^^10–12^^ which have been found to be most strongly and consistently associated with chronic widespread and regional pain in adulthood in previous studies.

#### 2.4.1. Early life socioeconomic and psychosocial environment

Parental levels of education were obtained from mothers' reports when participants were aged 6 years and categorised into 3 groups: secondary and higher; secondary only or primary and further education or higher; primary and further education (no qualifications attained) or primary only. Occupational class was assessed using father's occupation when the participant was 4 years old (where this was unknown [n = 65], father's occupation at age 11 or 15 years was used) and classified as high (I or II); middle (IIINM or IIIM); low (IV or V). Information on family disruption due to parental divorce before the study member was 15 years old came from mothers' reports during childhood and from study participants' reports when they were 26 years old.

#### 2.4.2. Sociodemographic factors during adulthood

The highest education level achieved by age 26 was coded using the Burnham classification and grouped into no qualifications, up to O level or equivalent, or A level or equivalent and above.^^6^^ Own occupation at age 53 (or if not available, the most recent measure in adulthood [n = 256]) was categorised according to Registrar General's social classification (Office of National Statistics (ONS), 1990) into 3 groups: high (I or II); middle (IIINM or IIIM); and low (IV or V).

#### 2.4.3. Adulthood health status and health behaviours

Body mass index was calculated from height (m) and weight (kg) measured at age 60 to 64 by a trained nurse during a clinical assessment at this age and classified into standard categories: <25, 25 to 29.9, and ≥30 kg/m^2^.

Symptoms of anxiety and depression were assessed at age 60 to 64 using the 28-item General Health Questionnaire (GHQ-28). Each item was coded using the General Scoring Method (ie, 0 for response choices 1 and 2; and as 1 or response choices 3 and 4), then summed, and a threshold for caseness of 5 or more was selected.^^9^^

At age 68, participants reported any long-standing illness or health problems which had lasted, or were expected to last for 6 months or more.

Smoking status was assessed by self-report across adulthood up to age 68 and categorised as never, ex-, and current smoker. Self-reported level of participation in sports, vigorous leisure activities, or exercises was assessed at age 68, and participants were grouped as inactive, less active (1-4 times/mo), or more active (≥5 times/mo).

### 2.5. Statistical analysis

Descriptive analyses including Pearson χ^2^ tests were used to assess the characteristics of study participants included in analyses.

As the dependent variable consisted of 4 categories (no pain, CWP, CRP, and other pain), multinomial logistic regression models were used to test the associations between serious illness and pain outcomes, with no pain as the referent category. Initial models were adjusted for sex, and formal tests of sex interaction performed. As there was no evidence of interaction, all subsequent models included men and women with adjustment made for sex. We then included each covariate, one at a time, in a sex-adjusted model of the association between serious illness and pain and those that were associated with pain in these models were included in subsequent models, grouped according to which of the 3 proposed pathways in Figure 1 (ie, socioeconomic; health status and behaviours; and psychosocial factors) they represented. Each group of factors was then adjusted for in turn before all 3 sets of factors were included in a fully adjusted model. All analyses were performed on the sample with complete data on both serious illness and pain (n = 2401). To reduce potential bias due to missing data, covariates with missing values (ie, own occupational class [n = 33], education [n = 124], BMI [n = 474], long-standing illness or health problems [n = 24], smoking [n = 24], physical activity [n = 27], and GHQ-28 [n = 499]) were imputed using multiple imputation by chained equations according to published guidelines.^^40^^ Analyses were performed across 20 imputed data sets and combined using Rubin rules.

Analyses were then repeated to assess the association of number of periods of serious illness with pain outcomes at age 68.

Sensitivity analyses examined serious illness broken down by type and complete case analysis was conducted to compare estimates obtained with those from the imputed data sets.

All analyses were performed using STATA version 14.0.

## 3. Results

At age 68, a total of 1339 (55.6%) participants reported pain, with a high proportion of participants reporting CRP (30.2%). Chronic widespread pain was more common in women than that in men (13.2% vs 7.7%) (Table 1).

**Table 1.**
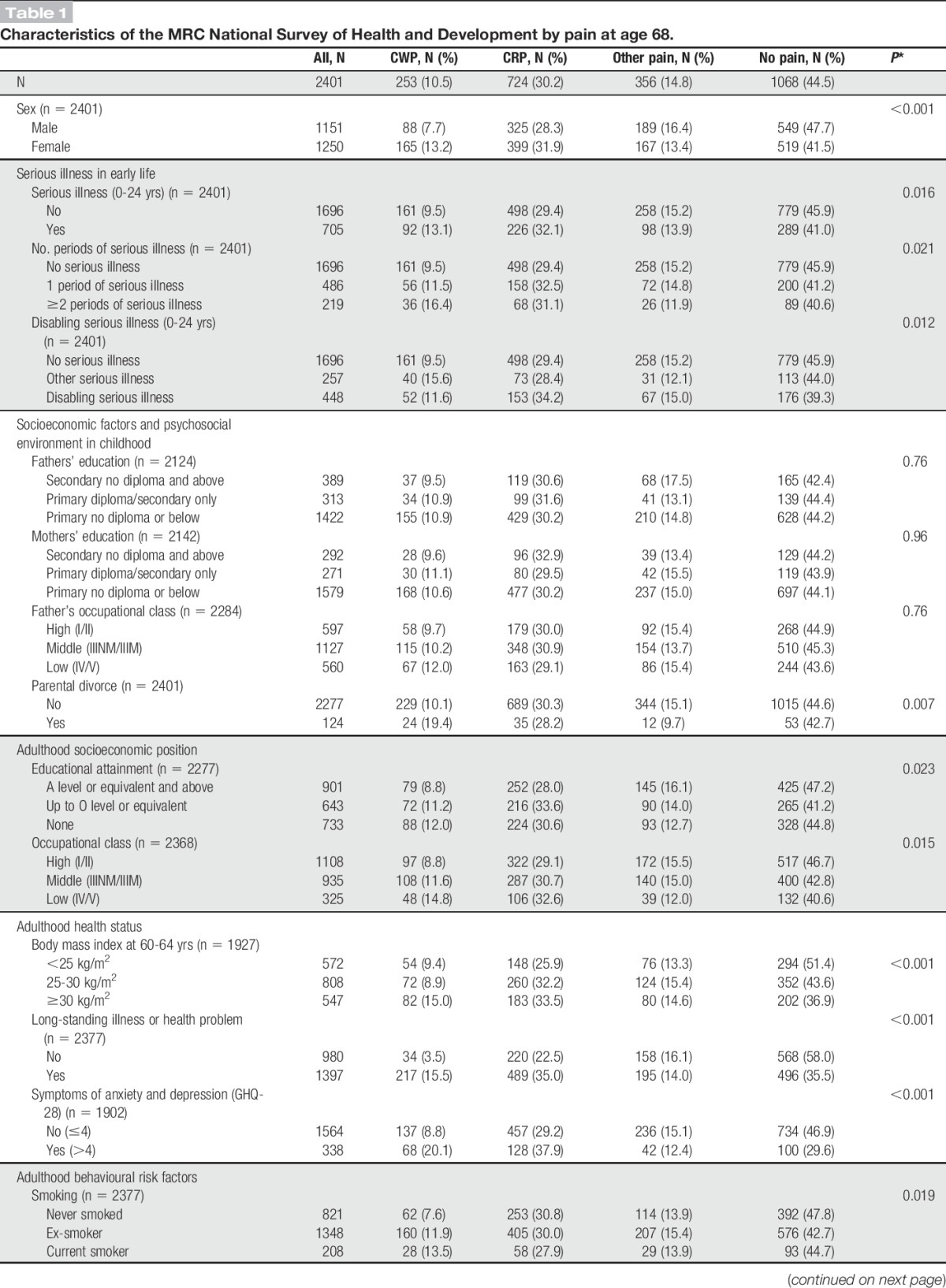

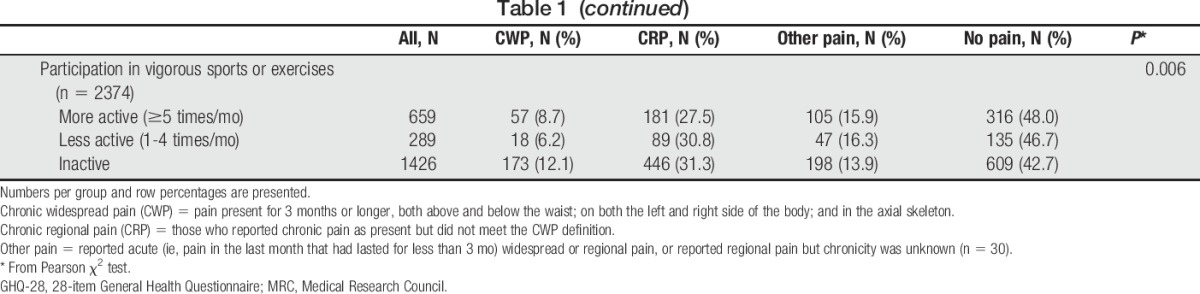
Characteristics of the MRC National Survey of Health and Development by pain at age 68.

Almost a third of all participants (29%) had experienced at least 1 serious illness before age 25; a higher proportion of boys than girls were affected (32% vs 27%, *P* = 0.003). A greater proportion of those who had a serious illness before age 25 had CWP compared with those who did not experience serious illness (13.1% vs 9.5%) (Table 1). Similarly, a high proportion of those who had a serious illness at 2 or more periods during early life had CWP compared with those who did not experience serious illness (16.4% vs 9.5%).

When compared with those not reporting pain, those who reported CWP were more likely to be obese, to be current smokers, to be physically inactive, to have a low occupational class and lower educational level, to have experienced parental divorce, and to report long-standing illness or health problems, and symptoms of anxiety and depression in adulthood (Table 1).

### 3.1. Associations between serious illness and pain outcomes

Findings from the sex-adjusted multinomial logistic regression models indicated that those who had experienced a serious illness before age 25 had a higher likelihood of CWP and of CRP vs no pain, compared with those with no history of serious illness (relative risk ratio [RRR] = 1.62 [95% CI: 1.21-2.17] and RRR = 1.25 [95% CI: 1.01-1.54], respectively). Adjustment for indicators of socioeconomic position slightly decreased these RRRs but did not alter the results significantly (Table 2, model 2). Adjustment for adult health status and health behaviours had a greater impact (Table 2, model 3), but there was little impact of adjustment for psychosocial factors (Table 2, model 4). In a fully adjusted model, associations with CWP were only partially attenuated; fully adjusted RRR = 1.43 (95% CI: 1.05-1.95) and 1.19 (95% CI: 0.96-1.48) for CWP and CRP, respectively (Table 2, model 5). No association was observed between serious illness and other pain.

**Table 2.**
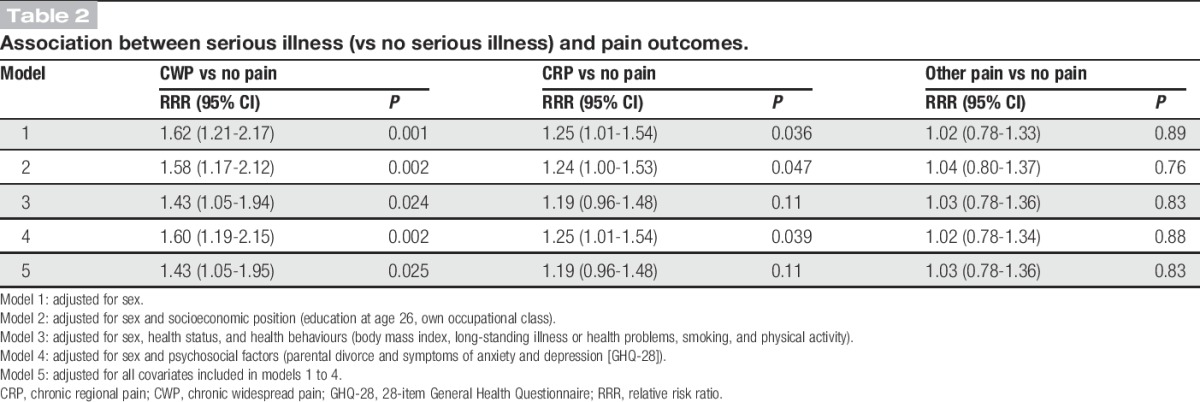
Association between serious illness (vs no serious illness) and pain outcomes.

There was some evidence of a cumulative association between serious illness and CWP (but not CRP or other pain); compared with those with no history of serious illness, the fully adjusted RRR for CWP was 1.25 (95% CI: 0.87-1.81) for those who had serious illness at least once, and 1.83 (95% CI: 1.16-2.88) for those who had serious illness 2 or more times (Table 3).

**Table 3.**
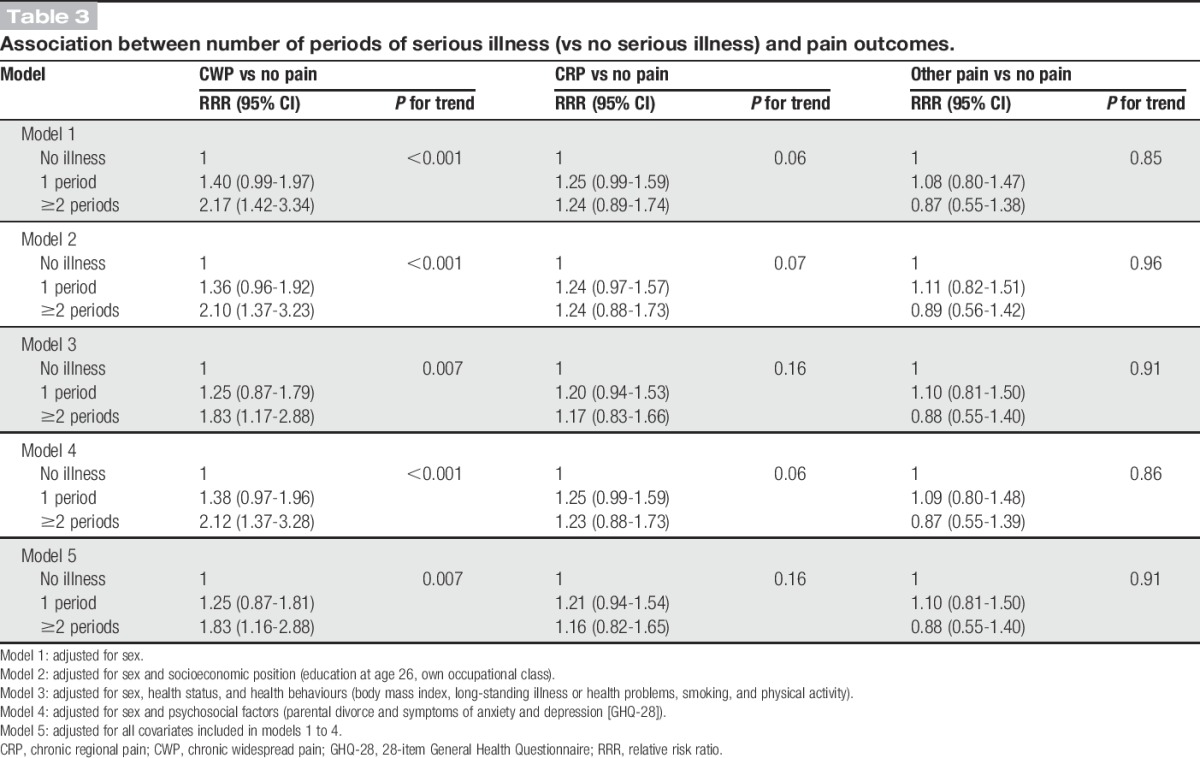
Association between number of periods of serious illness (vs no serious illness) and pain outcomes.

### 3.2. Sensitivity analysis

When we examined associations by type of serious illness (ie, potentially disabling or nondisabling serious conditions), there was some evidence that effect estimates varied slightly by type (Table S1, available online at http://links.lww.com/PAIN/A314), but when these differences were formally tested, there was no clear evidence that the strength of the associations with CWP differed (*P* = 0.25).

When analyses were restricted to those with complete data (n = 1731), we found that the overall patterns and trends of associations (Table S2, available online at http://links.lww.com/PAIN/A314) were similar to those from analyses using multiply imputed data sets, but the relationships were stronger, suggesting that excluding participants with incomplete data from the analyses would have been likely to overestimate the associations (comparing Table 2 and Table S2).

## 4. Discussion

### 4.1. Summary of main findings

In a large nationally representative British cohort followed up prospectively since birth, experience of serious illness before age 25 was associated with increased risk of CWP at age 68, even after adjustment for a range of potential confounders and mediators. There was evidence that this association was cumulative; those who experienced serious illness 2 or more times had the highest likelihood of CWP. Weaker associations found with CRP were fully attenuated after adjustments, and there was no evidence of association with other pain.

### 4.2. Comparison with previous studies

Our findings build on those from previous studies of younger populations examining the relationship between hospitalisation in early life and CWP. Prospective analyses using data from the 1958 British birth cohort examined a range of physically traumatic events before age 7 and demonstrated an increased likelihood of CWP at age 45 associated with hospitalisation after a road traffic accident, but not with surgical operations and hospitalisations for other reasons (accidents at home/other accidents).^^14^^ Our study has considered a wider range of serious illnesses which were prospectively ascertained across a longer period in earlier life and were found to be associated with CWP in old age.

Our findings of weaker associations with CRP and no associations with other pain are consistent with previous NSHD findings^^11,12^^ but not findings from some other studies.^^18^^ Hotopf et al^^12^^ reported no associations between any serious physical illness before age 15 and chest pain or multiple medically unexplained physical symptoms (including these health complaints: rheumatism and arthritis, headache, backache and sciatica, abdominal pain, chest pain, and dizziness)^^11^^ at age 36 years in the NSHD. In contrast, a Canadian study of adults aged 18 years or older at baseline, which retrospectively assessed specific stressful experiences in childhood, found that hospitalisation for at least 2 weeks in childhood was associated with an increased likelihood of developing chronic back pain (a commonly reported CRP) after 4 years of follow-up.^^18^^

In the 1958 British birth cohort, Jones et al^^15^^ 2007 found that those who had multiple common symptoms (ie, periodic vomiting or bilious attacks, abdominal pain, or frequent headaches and migraines) before age 16 years had a higher likelihood of CWP at 45 years. However, previous work in NSHD found no association between persistent abdominal pain before age 15 years and persistent physical symptoms such as abdominal pain or headaches at age 36.^^10^^ Our analyses highlight that real differences may exist with inconsistencies in findings between studies possibly explained by a number of factors including variation in the definitions and assessment of childhood physical illness, illness severity and duration, definitions of pain used, or age at pain assessment. For example, that we have found that serious illness in earlier life was associated with pain in later life suggests that our broad definition of childhood chronic physical illnesses reflects a slightly different exposure to that used in some other articles which examined chronic illness due to specific diagnoses.^^33^^

### 4.3. Explanation of findings

There was little impact on effect sizes of the associations of serious illness with CWP or CRP when socioeconomic or psychosocial factors were included in multivariable analysis, implying that the observed associations did not seem to be explained by these hypothesised pathways (Fig. 1). However, adult health status and adult health behaviours did attenuate the size of associations. This is consistent with findings from previous studies that have shown that higher BMI,^^2,26,42^^ smoking,^^37,42^^ chronic disease,^^28^^ and reduced levels of physical activity^^5,27^^ are associated with prevalent and development of chronic pain. However, associations of adult health status and health behaviours with pain may operate in both directions. For instance, McBeth et al^^27^^ found that individuals aged between 20 and 65 years who reported having some pain or CWP at baseline had increased likelihood of low self-reported levels of physical activity after 32 months of follow-up, suggesting that physical inactivity may be a consequence of CWP.^^27^^ In addition, some reports have demonstrated that individuals with CRP have an increased likelihood of developing CWP,^^22^^ and a history of 2 or more painful comorbidities, moderate to severe pain intensity and a high number of reported painful regions at baseline are also important predictors for the development and persistence of CWP at follow-up.^^3,17^^

We found stronger associations of serious illness in earlier life with CWP than with CRP and no associations with other pain which may suggest different underlying mechanisms. A plausible explanation is that CWP is not an independent entity but rather falls within a pain-distress continuum, with somatic distress and symptoms as one feature of CWP.^^3,17,43^^ Other studies have demonstrated that CWP may co-occur with chronic syndromes that are frequently unexplained (eg, irritable bowel syndrome, chronic orofacial pain, and chronic fatigue) further supporting the notion that CWP probably shares similar underlying mechanisms, eg, central sensitisation with these disorders.^^1,17^^ As the association of serious illness in earlier life with CWP in later life was not fully explained by a wide range of lifetime factors in our study, other potential pathways such as those related to fear-avoidance beliefs^^23,44^^ may partly explain our findings, and so require further examination in data sets with relevant data.

### 4.4. Methodological considerations

Our study has a number of major strengths, including the large sample of older adults, prospective collection of information on hospital admission across all stages of early life, and a range of potential confounders across life. Reports on hospitalisation were checked with the relevant hospital for details of the admission, removing the potential of recall bias. We compared different pain subgroups to test whether serious illness was differentially associated with CWP, CRP, and other pain.

One limitation is that pain was assessed at a single time point. Thus, we were not able to discern whether some of the covariates preceded the onset of pain outcomes which may have led to overadjustment in our models. Similarly, we were unable to examine whether there were differences in association by age at onset of pain. For example, we were unable to assess whether those people who experienced serious illness in earlier life had experienced pain throughout their adult lives, or whether they were at higher risk of developing chronic pain in later life. A second limitation is that our findings could be explained by residual confounding; some covariates, eg, leisure time physical activity, included in the analysis were assessed at one time point and so may not fully reflect lifetime exposure. There are other aspects of behavioural and psychosocial risk, in addition to those we selected a priori and included in our models, which may be important. However, in follow-up analyses when we adjusted for a cumulative leisure time physical activity score, this had little impact on our findings (results not shown). A third limitation is that those who experienced serious illness in early life were underrepresented in the study sample which may have introduced survival bias. In additional analyses, we found that participants who had experienced serious illness in earlier life had higher rates of death during follow-up when compared with those with no history of serious illness (hazard ratio = 1.38 [95% CI: 1.15-1.65]), after adjustment for sex, father's and own occupational class. However, families with children with serious illness were less likely to emigrate in this cohort.^^7^^

As with all longitudinal studies, there has also been avoidable loss to follow-up through loss of contact and permanent refusal, but there were no differences by childhood serious illness. The NSHD has remained broadly representative of the population born at a similar time in most key characteristics, although those still participating at age 60 to 64 had higher rates of house ownership and lower rates of long-term limiting illness than the equivalent general population in England.^^39^^ This may limit the generalisability of these findings to more recent cohorts and ethnically diverse cohorts. The prevalence of some chronic childhood illnesses (eg, asthma, diabetes, cancer, and mental disorders) and obesity has increased in recent decades,^^13,41^^ but there has been a marked decline in the rates of other types of serious illnesses (eg, polio and rheumatic heart disease). Nevertheless, older birth cohorts remain important resources for examining the long-term consequences of serious illnesses in earlier life on health-related outcomes in old age, and their findings can be used to help inform early life interventions and health policies for future generations of older people.

## 5. Conclusions

Findings from this large British birth cohort study show that experience of serious illness in earlier life is associated with higher risk of CWP at age 68. This association is robust to adjustments and seems to be cumulative with those who had experienced more periods of illness at the highest risk of CWP. This suggests that those who have experienced serious illness in earlier life, especially those who have experienced multiple episodes, may require more support across life than others to minimise their risk of CWP in later life.

## Conflict of interest statement

The authors have no conflicts of interest to declare.

This work was supported by the UK Medical Research Council which provides core funding for the MRC National Survey of Health and Development and supports S. G. Muthuri, D. Kuh and R. Cooper (programme codes: MC_UU_12019/1 and MC_UU_12019/4). S. G. Muthuri is also supported by MRC grant MR/L010399/1. R. Bendayan is supported by the National Institute on Aging of the National Institutes of Health under award number P01AG043362.

Data used in this publication are available to bona fide researchers upon request to the NSHD Data Sharing Committee via a standard application procedure. Further details can be found at http://www.nshd.mrc.ac.uk/data. doi: 10.5522/NSHD/Q101; doi: 10.5522/NSHD/Q102; 10.5522/NSHD/Q103.

## Appendix A Supplemental Digital Content

Supplemental Digital Content associated with this article can be found online at http://links.lww.com/PAIN/A314.

## References

[1] Aggarwal VR McBeth J Zakrzewska JM Lunt M Macfarlane GJ The epidemiology of chronic syndromes that are frequently unexplained: do they have common associated factors? Int J Epidemiol 2006;35:468–76. 16303810 10.1093/ije/dyi265

[2] Antony B Jones G Venn A Cicuttini F March L Blizzard L Dwyer T Cross M Ding C Association between childhood overweight measures and adulthood knee pain, stiffness and dysfunction: a 25-year cohort study. Ann Rheum Dis 2015;74:711–17. 24347570 10.1136/annrheumdis-2013-204161

[3] Bergman S Herrstrom P Jacobsson LTH Petersson IF Chronic widespread pain: a three year followup of pain distribution and risk factors. J Rheum 2002;29:818–25. 11950027

[4] Blackwell DL Hayward MD Crimmins EM Does childhood health affect chronic morbidity in later life? Soc Sci Med 2001;52:1269–84. 11281409 10.1016/s0277-9536(00)00230-6

[5] Dansie EJ Turk DC Martin KR Van Domelen DR Patel KV Association of chronic widespread pain with objectively measured physical activity in adults: findings from the national health and nutrition examination survey. J Pain 2014;15:507–15. 24462501 10.1016/j.jpain.2014.01.489

[6] Department of Education and Science. Burnham Further Education Committee Grading Courses. London: HMSO, 1972.

[7] Fuller-Thomson E Brennenstuhl S Cooper R Kuh D An investigation of the healthy migrant hypothesis: pre-emigration characteristics of those in the British 1946 birth cohort study. Can J Public Health 2016;106:e502–8. 26986911 10.17269/CJPH.106.5218 PMC6972096

[8] Gardener EA Huppert FA Guralnik JM Melzer D Middle-aged and mobility-limited: prevalence of disability and symptom attributions in a national survey. J Gen Intern Med 2006;21:1091–6. 16970558 10.1111/j.1525-1497.2006.00564.x PMC1831629

[9] Goldberg DP Hillier VF A scaled version of the general health questionnaire. Psychol Med 1979;9:139–45. 424481 10.1017/s0033291700021644

[10] Hotopf M Carr S Mayou R Wadsworth M Wessely S Why do children have chronic abdominal pain, and what happens to them when they grow up? Population based cohort study. BMJ 1998;316:1196–200. 9552994 10.1136/bmj.316.7139.1196 PMC28520

[11] Hotopf M Mayou R Wadsworth M Wessely S Childhood risk factors for adults with medically unexplained symptoms: results from a national birth cohort study. Am J Psychiatry 1999;156:1796–800. 10553745 10.1176/ajp.156.11.1796

[12] Hotopf M Mayou R Wadsworth M Wessely S Psychosocial and developmental antecedents of chest pain in young adults. Psychosom Med 1999;61:861–7. 10593639 10.1097/00006842-199911000-00022

[13] Johnson W Li L Kuh D Hardy R How has the age-related process of overweight or obesity development changed over time? Co-ordinated analyses of individual participant data from five United Kingdom birth cohorts. PLos Med 2015;12:e1001828. 25993005 10.1371/journal.pmed.1001828 PMC4437909

[14] Jones GT Power C Macfarlane GJ Adverse events in childhood and chronic widespread pain in adult life: results from the 1958 British Birth Cohort Study. PAIN 2009;143:92–6. 19304391 10.1016/j.pain.2009.02.003

[15] Jones GT Silman AJ Power C Macfarlane GJ Are common symptoms in childhood associated with chronic widespread body pain in adulthood? Results from the 1958 British Birth Cohort Study. Arthritis Rheum 2007;56:1669–75. 17469161 10.1002/art.22587

[16] Kamphuis M Ottenkamp J Vliegen HW Vogels T Zwinderman KH Kamphuis RP Verloove-Vanhorick SP Health related quality of life and health status in adult survivors with previously operated complex congenital heart disease. Heart 2002;87:356–62. 11907011 10.1136/heart.87.4.356 PMC1767074

[17] Kindler LL Jones KD Perrin N Bennett RM Risk factors predicting the development of widespread pain from chronic back or neck pain. J Pain 2010;11:1320–8. 20488762 10.1016/j.jpain.2010.03.007 PMC2950865

[18] Kopec JA Sayre EC Stressful experiences in childhood and chronic back pain in the general population. Clin J Pain 2005;21:478–83. 16215332 10.1097/01.ajp.0000139909.97211.e1

[19] Kuh D Pierce M Adams J Deanfield J Ekelund U Friberg P Ghosh AK Harwood N Hughes A Macfarlane PW Mishra G Pellerin D Wong A Stephen AM Richards M Hardy R Cohort Profile: updating the cohort profile for the MRC National Survey of Health and Development: a new clinic-based data collection for ageing research. Int J Epidemiol 2011;40:e1–9. 21345808 10.1093/ije/dyq231 PMC3043283

[20] Kuh DJ Wadsworth ME Yusuf EJ Burden of disability in a post war birth cohort in the UK. J Epidemiol Community Health 1994;48:262–9. 8051525 10.1136/jech.48.3.262 PMC1059957

[21] Kuh DJL Wadsworth MEJ Physical health status at 36 years in a British national birth cohort. Soc Sci Med 1993;37:905–16. 8211309 10.1016/0277-9536(93)90145-t

[22] Larsson B Bjork J Borsbo B Gerdle B A systematic review of risk factors associated with transitioning from regional musculoskeletal pain to chronic widespread pain. Eur J Pain 2012;16:1084–93. 22362638 10.1002/j.1532-2149.2012.00117.x

[23] Leeuw M Goossens MEJB Linton SJ Crombez G Boersma K Vlaeyen JWS The fear-avoidance model of musculoskeletal pain: current state of scientific evidence. J Behav Med 2006;30:77–94. 17180640 10.1007/s10865-006-9085-0

[24] Leveille SG Bean J Ngo L McMullen W Guralnik JM The pathway from musculoskeletal pain to mobility difficulty in older disabled women. PAIN 2007;128:69–77. 17055167 10.1016/j.pain.2006.08.031 PMC2555988

[25] Leveille SG Fried L Guralnik JM Disabling symptoms: what do older women report? J Gen Intern Med 2002;17:766–73. 12390552 10.1046/j.1525-1497.2002.20229.x PMC1495119

[26] Macfarlane GJ de Silva V Jones GT The relationship between body mass index across the life course and knee pain in adulthood: results from the 1958 birth cohort study. Rheumatology 2011;50:2251–6. 21984765 10.1093/rheumatology/ker276

[27] McBeth J Nicholl BI Cordingley L Davies KA MacFarlane GJ Chronic widespread pain predicts physical inactivity: results from the prospective EPIFUND study. Eur J Pain 2010;14:972–9. 20400346 10.1016/j.ejpain.2010.03.005 PMC3161181

[28] Mundal I Grawe RW Bjorngaard JH Linaker OM Fors EA Prevalence and long-term predictors of persistent chronic widespread pain in the general population in an 11-year prospective study: the HUNT study. BMC Musculoskelet Disord 2014;15:213. 24951013 10.1186/1471-2474-15-213 PMC4089927

[29] Oeffinger KC Mertens AC Sklar CA Kawashima T Hudson MM Meadows AT Friedman DL Marina N Hobbie W Kadan-Lottick NS Schwartz CL Leisenring W Robison LL Chronic health conditions in adult survivors of childhood cancer. N Engl J Med 2006;355:1572–82. 17035650 10.1056/NEJMsa060185

[30] Patel KV Guralnik JM Dansie EJ Turk DC Prevalence and impact of pain among older adults in the United States: findings from the 2011 national health and aging trends study. PAIN 2013;154:2649–57. 24287107 10.1016/j.pain.2013.07.029 PMC3843850

[31] Platt OS Brambilla DJ Rosse WF Milner PF Castro O Steinberg MH Klug PP Mortality in Sickle Cell disease—life expectancy and risk factors for early death. N Engl J Med 1994;330:1639–44. 7993409 10.1056/NEJM199406093302303

[32] Pless IB Cripps HA Davies JMC Wadsworth MEJ Chronic physical illness in childhood: psychological and social effects in adolescence and adult life. Dev Med Child Neurol 1989;31:746–55. 2599268 10.1111/j.1469-8749.1989.tb04070.x

[33] Pless IB Nolan T Revision, replication and neglect—research on maladjustment in chronic illness. J Child Psychol Psychiatry 1991;32:347–65. 2033113 10.1111/j.1469-7610.1991.tb00312.x

[34] Pless IB Power C Peckham CS Long-term psychosocial sequelae of chronic physical disorders in childhood. Pediatrics 1993;91:1131–6. 8502515

[35] Power C Peckham C Childhood morbidity and adulthood ill health. J Epidemiol Community Health 1990;44:69–74. 2348153 10.1136/jech.44.1.69 PMC1060601

[36] Sears MR Greene JM Willan AR Wiecek EM Taylor DR Flannery EM Cowan JO Herbison GP Silva PA Poulton R A longitudinal, population-based, cohort study of childhood asthma followed to adulthood. N Engl J Med 2003;349:1414–22. 14534334 10.1056/NEJMoa022363

[37] Shiri R Karppinen J Leino-Arjas P Solovieva S Viikari-Juntura E The association between smoking and low back pain: a meta-analysis. Am J Med 2010;123:87.e7–35. 20102998 10.1016/j.amjmed.2009.05.028

[38] Simmonds NJ Ageing in cystic fibrosis and long-term survival. Paediatr Respir Rev 2013;14(suppl 1):6–9. 23497942 10.1016/j.prrv.2013.01.007

[39] Stafford M Black S Shah I Hardy R Pierce M Richards M Wong A Kuh D Using a birth cohort to study ageing: representativeness and response rates in the National Survey of Health and Development. Eur J Ageing 2013;10:145–57. 23637643 10.1007/s10433-013-0258-8 PMC3637651

[40] Sterne JAC White IR Carlin JB Spratt M Royston P Kenward MG Wood AM Carpenter JR Multiple imputation missing data epidemiological clinical research: potential pitfalls. BMJ 2009;338:b2393. 19564179 10.1136/bmj.b2393 PMC2714692

[41] Van Cleave J Gortmaker SL Perrin JM Dynamics of obesity and chronic health conditions among children and youth. JAMA 2010;303:623–30. 20159870 10.1001/jama.2010.104

[42] VanDenKerkhof EG Macdonald HM Jones GT Power C Macfarlane GJ Diet, lifestyle and chronic widespread pain: results from the 1958 British birth cohort study. Pain Res Manag 2011;16:87–92. 21499583 10.1155/2011/727094 PMC3084409

[43] Viniol A Jegan N Brugger M Leonhardt C Barth J Baum E Becker A Strauch K Even worse—risk factors and protective factors for transition from chronic localized low back pain to chronic widespread pain in general practice: a cohort study. Spine 2015;40:E890–9. 25955187 10.1097/BRS.0000000000000980

[44] Vlaeyen JWS Linton SJ Fear-avoidance model of chronic musculoskeletal pain: 12 years on. PAIN 2012;153:1144–7. 22321917 10.1016/j.pain.2011.12.009

[45] Wadsworth M Kuh D Richards M Hardy R Cohort profile: the 1946 national birth cohort (mrc national survey of health and development). Int J Epidemiol 2006;35:49–54. 16204333 10.1093/ije/dyi201

[46] Wolfe F Smythe HA Yunus MB Bennett RM Bombardier C Goldenberg DL Tugwell P Campbell SM Abeles M Clark P Fam AG Farber SJ Fiechtner JJ Michael Franklin C Gatter RA Hamaty D Lessard J Lichtbroun AS Masi AT McCain GA John Reynolds W Romano TJ Jon Russell I Sheon RP The American College of Rheumatology 1990 criteria for the classification of fibromyalgia. Arthritis Rheum 1990;33:160–72. 2306288 10.1002/art.1780330203

